# Evodiamine induces ferroptosis in prostate cancer cells by inhibiting TRIM26-mediated stabilization of GPX4

**DOI:** 10.1186/s13020-025-01130-0

**Published:** 2025-05-26

**Authors:** Lanlan Li, Jianzhong Lu, Shengjun Fu, Wenyan Li, Ying Wang, Ke Wang, Yan Tao, Shanhui Liu

**Affiliations:** 1 Institute of Urology, Gansu Province Clinical Research Center for Urinary System Diseases, Lanzhou University Second Hospital, No. 82 Cuiyingmen, Lanzhou, 730030 Gansu China; 2 Department of Gynaecology and Obstetrics, Lanzhou University Second Hospital, No. 82 Cuiyingmen, Lanzhou, 730030 Gansu China; 3 The Second Hospital & Clinical Medical School, Lanzhou University, Lanzhou, 730030 Gansu China

**Keywords:** Evodiamine, Prostate cancer, Ferroptosis, GPX4, TRIM26

## Abstract

**Background:**

Prostate cancer is a major global health challenge, characterized by high morbidity and mortality rates. Traditional treatment options, including androgen deprivation therapy and chemotherapy, often lead to drug resistance. In recent years, natural compounds have garnered attention for their potential therapeutic effects. Evodiamine, a bioactive alkaloid from Evodia rutaecarpa, has demonstrated promising anti-cancer properties in various malignancies, including oral squamous cell carcinoma, breast, colorectal, and ovarian cancers. This study explores the efficacy of evodiamine in prostate cancer cells and investigates the mechanisms underlying evodiamine-induced cell death.

**Methods:**

To investigate the effects of evodiamine on prostate cancer cells, various cell lines, including both castration-sensitive and castration-resistant variants, were treated with different concentrations of evodiamine for various durations. Cell viability, proliferation, invasion ability, and colony formation were assessed using the CCK8 assay, EdU assay, 3D matrigel drop invasion assay, and colony formation assay, respectively. The effects of evodiamine on apoptosis were analyzed using FACS, Hoechst staining, and Western blot. To evaluate its effects on ferroptosis, malondialdehyde (MDA) and glutathione (GSH) assay kits, as well as DCFH-DA and the lipid peroxidation sensor BODIPY^™^ 581/501 C11 fluorescent probes, were employed. The molecular mechanisms through which evodiamine regulates GPX4 protein instability were investigated using Western blot and TRIM26 ectopic expression. Additionally, a mouse xenograft model derived from DU145 cells was established to evaluate the in vivo effects of evodiamine and its molecular mechanisms, utilizing hematoxylin and eosin (H&E) staining, immunohistochemistry (IHC), and Western blot analysis.

**Results:**

Evodiamine significantly suppressed cell viability, proliferation, invasion, and colony formation in prostate cancer cells. Importantly, evodiamine-induced cell death in the PC3 and DU145 cell lines was independent of apoptosis pathway. Instead, evodiamine increased reactive oxygen species (ROS) production, lipid ROS levels and MDA levels, while decreasing GSH levels, indicating the induction of ferroptosis. The key role of ROS in evodiamine-induced ferroptosis was further confirmed by the partial reversal of cell death upon treatment with the ROS scavenger N-acetylcysteine (NAC). Mechanistically, evodiamine induced ferroptosis by destabilizing GPX4 protein in a TRIM26-dependent manner. Moreover, in vivo studies demonstrated that evodiamine significantly inhibited tumor growth and induced ferroptosis in tumor cells, highlighting its therapeutic potential.

**Conclusion:**

This study demonstrates that evodiamine exerts potent antitumor effects against prostate cancer through inhibiting TRIM26-mediated stabilization of GPX4 protein and triggering ferroptosis. These findings suggest that evodiamine, a natural product derived from traditional Chinese medicine, could be a promising therapeutic agent for prostate cancer.

**Graphical Abstract:**

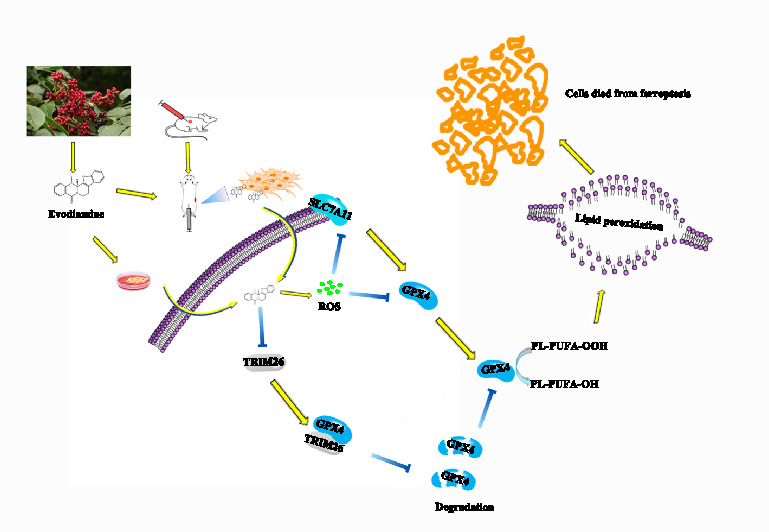

**Supplementary Information:**

The online version contains supplementary material available at 10.1186/s13020-025-01130-0.

## Introduction

Prostate cancer (PCa) remains a significant global health issue, characterized by its high incidence and substantial mortality rates [1, 2]. Despite the advancements in traditional treatment modalities, such as androgen deprivation therapy (ADT) and chemotherapy, the development of drug resistance, particularly in castration-resistant prostate cancer (CRPC), continues to be a major challenge [3]. Drug resistance mechanisms in PCa include alterations in androgen receptor signaling, activation of alternative growth pathways, and enhanced cellular survival strategies [4–6]. Consequently, there is an urgent need for novel therapeutic strategies to overcome resistance and improve clinical outcomes.

In recent years, ferroptosis, a form of iron-dependent regulated cell death characterized by lipid peroxidation and cellular iron overload, has gained attention as a therapeutic target in cancer treatment [7, 8]. Unlike apoptosis, which involves cell death through intrinsic or extrinsic pathways, ferroptosis is mediated by the accumulation of lipid peroxides and the depletion of GSH. Inducing ferroptosis has been proposed as a strategy to circumvent drug resistance in various cancers, presenting a potential avenue for overcoming the limitations of current therapies [9, 10]. The ability of ferroptosis inducers to target cancer cells with high iron content and altered redox states presents an intriguing opportunity for enhancing treatment efficacy.

Recent research has highlighted the potential of natural compounds as alternative or adjunct therapies for cancer treatment, including PCa [11]. Among these compounds, evodiamine (evod), a bioactive alkaloid extracted from Evodia rutaecarpa (Wu Zhu Yu)—a traditional Chinese herb historically used to treat headaches, postpartum hemorrhage, and gastrointestinal disorders, with emerging evidence supporting its anticancer properties [12–15]. This phytochemical has exhibited remarkable antitumor efficacy across multiple cancer types, including oral squamous cell carcinoma [16, 17], breast cancer [18, 19], colorectal cancer [20], and ovarian cancer [21]. The therapeutic efficacy of evodiamine in PCa, especially in CRPC, is of particular interest, as it may offer a novel approach to treatment by targeting mechanisms beyond traditional therapies. Numerous studies demonstrated that evodiamine inhibits cancer cells growth via the induction of apoptosis and cell cycle arrest [21–23]. Recent studies suggested its anti-cancer activity potential via activating ferroptosis pathway [24]. In addition to its ability to directly induce cell death, evodiamine may act as a promising agent in either PCa anti-angiogenic therapy or immunotherapy by inhibiting Sema3A-mediated angiogenesis and PD-L1 expression [12]. Although numerous studies have demonstrated the anticancer efficacy of evodiamine across diverse pathways, the precise mechanisms underlying its action, particularly its capacity to activate the ferroptosis pathway, remain elusive.

The present study focuses on evaluating the anti-cancer effects of evodiamine in PCa cells, with a particular emphasis on its role in inducing ferroptosis. Using a range of cell lines, including both castration-sensitive and castration-resistant, this work aims to elucidate the mechanisms by which evodiamine affects cell viability, proliferation, and invasion. We found that evodiamine induced prostate cancer cells ferroptosis mainly depending on suppressing the stability of the GPX4 protein. We also found that TRIM26, an E3 ubiquitin ligase that enhances the stability of the GPX4 protein, is down-regulated in expression upon stimulation by evodiamine. These results suggested that evodiamine may induce prostate cancer cells ferroptosis via inhibiting TRIM26-mediated GPX4 protein stability.

In summary, this study aims to enhance the understanding of evodiamine's therapeutic potential in PCa. It deepens our understanding of how evodiamine induces ferroptosis, revealing for the first time that evodiamine can induce ferroptosis by inhibiting TRIM26-mediated GPX4 protein stability. The findings will provide valuable insights into the application of traditional Chinese medicine-derived compounds in modern cancer therapy and provide a new therapeutic strategy against PCa.

## Materials and methods

### Cell culture

LNCaP, 22RV1, VCaP, PC3 and DU145 cells were obtained from the Chinese Academy of Sciences Cell Bank of Type Culture Collection. All cell lines were cultured in RPMI 1640 medium, F-12 K medium or Dulbecco’s modified Eagle’s medium supplemented with 10% fetal bovine serum and 1% penicillin/streptomycin and incubated at 37 °C under 5% CO_2_.

### Chemicals and antibodies

Evodiamine (T2868), z-VAD-fmk (T7020), Bafilomycin A1 (T6740), Ferrostatin-1 (T6500), Deferoxamine Mesylate (T1637) and puromycin (T19978) were purchased from Topscience. CCK8 reagent (CYT001) was obtained from Yoche. Hoechst 33258 (C1018), N-Acetyl-L-cysteine (ST2524), Reactive Oxygen Species Assay Kit (S0033S), Lipid Peroxidation MDA Assay Kit (S0131S), Total Glutathione Peroxidase Assay Kit with NADPH (S0058), and Annexin V-FITC Apoptosis Detection Kit (C1062L) were purchased from Beyotime. MG132 (HY-13259) was obtained from MedChem Express. BODIPY™ 581/591 C11 (D3861) was sourced from Thermo Fisher Scientific. Matrigel (356234) was obtained from CORNING.

The primary antibodies used for IHC and Western blot were following: GAPDH (ab128915), γH2AX (ab124781), caspase-9 (ab32539) and BAX (ab32503) were purchased from abcam. FSP1 (A12128), SLC40A1 (A14885), FTH1 (A19544), FTL (A1768), DMT1 (A10231), CD71 (A5865), SLC7A11/xCT (A2413), GPX4 (A1933), SQSTM1/p62 (A21702), LC3B (A19665), TRIM26 (A15332), USP7 (A13564), USP25 (A12588), USP14 (A16643), OTUB1 (A11656), USP10 (A7505), HSC70 (A0415) and Ki67 (A21861) were purchased from Abclonal. NCOA4 (DF4255) was purchased from affinity. BCL2 (2872), PARP (9532), cleaved-caspase3 (9664S) and BAK (12105) were purchased from Cell Signaling Technology. IRDye^®^ 680RD Goat anti-Mouse IgG secondary antibody and IRDye^®^ 800CW Goat anti-Rabbit IgG secondary antibody were purchased from LI-COR.

### Plasmids construction and transfection

ASLC7A11, GPX4 and FSP1 overexpression vector were constructed by Miaolingbio (Wuhan, China). Lentivirus particles are produced by transfecting the HEK293T cells with the overexpression vectors, packaging plasmid psPAX.2, and the envelope plasmid pMD2.G. The virus-containing supernatant was harvested at 48 h after transfection. Prostate cancer cell lines were infected with the lentiviral supernatants in the presence of polybrene for 12 h and were subsequently selected with puromycin (8 μg/ml).

### Cells viability assay

Cells were seeded into 96-well plates and treated with varying concentrations of evodiamine, NAC, or DMSO for either 24 or 48 h. After the respective treatment period, CCK8 reagent was added to each well and incubated at 37 ℃ for 2–4 h. Subsequently, the absorbance at 450 nm was measured by using a microplate reader.

### Clonogenic assays

The colony-forming-unit (CFU) assay was conducted to assess the effect of evodiamine on the indicated cells. Briefly, the cells were treated with various concentrations of evodiamine or DMSO. Media and evodiamine were refreshed every two days for duration of ten days. Subsequently, the formed colonies were fixed with 4% paraformaldehyde and stained with a crystal violet solution. The number of colonies was quantified for each sample.

### Cell invasion assay

Cell invasion ability was evaluated using the 3D matrigel drop invasion assay. Briefly, 5 × 10^4^ cells were mixed with 10 μl of matrigel and pipetted as droplets into a 24-well plate, allowing them to solidify for 20 min before adding media. The formed tumoroids were then treated with either DMSO or evodiamine for a duration of seven days, with media and evodiamine replaced every two days [10].

### PI/annexinV staining analyses

Cells were plated in 60 mm dishes and treated with DMSO or evodiamine for a duration of 48 h. Subsequently, the cells were collected and stained with Annexin V-FITC apoptosis kit according to the manufacturer’s instructions. Flow cytometry (BECKMAN CytoFLEX) was employed to detect and quantify the fluorescence. All flow data were analyzed with FlowJo 10.8.1.

### EdU thymidine incorporation assay

Click-iT EdU cell proliferation assays was conducted to assess DNA synthesis ability of prostate cancer cells. The proliferating cells were labeled with BeyoClick^™^ EdU Cell Proliferation Kit with Alexa Fluor 488 according to the manufacturer's protocol. Fluorescence was also visualized using a fluorescence microscope.

### Intracellular reactive oxygen species assays

Intracellular ROS levels were measured using the fluorescent probe DCFH-DA according to previously described protocols. Briefly, the stimulated prostate cancer cell lines were exposed to 25 μM DCFH-DA for 30 min in the dark. Then, the fluorescence was then monitored using flow cytometry (BECKMAN CytoFLEX) to quantify intracellular ROS levels.

### Determination of lipid ROS

Cellular lipid ROS level was determined using C11-BODIPY581/591 staining, following the manufacturer's instructions. Cells were washed with phosphate-buffered saline (PBS) and incubated with 2 µM solution of the C11-BODIPY581/591 fluorescent probe for 15 min at room temperature in the dark. Subsequently, cells were analyzed using a fluorescence microscope.

### Malondialdehyde and total Glutathione Peroxidase assay

PC3 and DU145 cells were seeded in 100 mm dishes and treated with either DMSO or evodiamine for 48 h. Subsequently, the levels of MDA and total glutathione peroxidase were quantified using the Lipid Peroxidation MDA Assay Kit and the Total Glutathione Peroxidase Assay Kit, following the manufacturer’s instructions.

### Quantitative RT-PCR (qRT-PCR)

mRNA expression analysis was performed following a previously described protocol [25]. Briefly, total RNAs were isolated from indicated prostate cancer cells using TRIzol reagent. Subsequently, the extracted RNA was converted into cDNA using the SweScript RT II First Strand cDNA Synthesis Kit (With gDNA Remover). Target gene quantification was performed using the 2 × SYBR Green qPCR Master Mix and the Bio-Rad CFX-96 thermal cycler. The mRNA expression levels of the target gene were normalized to β-actin. The primer sequences used were as follows: *gpx4* (forward:5ʹ-atcctggccttcccgtgtaac-3ʹ; reverse:5ʹ-cttgcccttgggttggatctt-3ʹ); *slc7a11* (forward:5ʹ-accatcattggagcaggaa-3ʹ; reverse:5ʹ-acacaccgtccagatggtc-3ʹ); *fsp1* (forward:5ʹ-attggtgactgtgccgacgtg-3ʹ; reverse:5ʹ-gatttggcccacaccgtcatt-3ʹ); *trim26* (forward:5ʹ-gggaccctgtgaccattgact-3ʹ; reverse:5ʹ-ccacacgggtcggatgttc-3ʹ); *β-actin* (forward: 5ʹ-gggaaatcgtgcgtgacatt-3ʹ; reverse:5ʹ-ggaaccgctcattgccaat-3ʹ).

### Western blot analysis

Cells were treated as indicated and subsequently lysed in the cell lysis buffer supplemented with protease /phosphatase inhibitor cocktail. The protein concentration in the lysate was quantified using the bicinchoninic acid (BCA) kit. Following quantification, the supernatants were subjected to 12% SDS-PAGE, and the proteins were transferred onto PVDF membranes for detection of P-γH2AX, caspase9, PARP, caspase3, BCL2, BAK, BAX, SLC7A11, GPX4, FSP1, NAOA4, FTH, CD71, SLC40A1, P62, FTL, DMT1, LC3B, OTUB1, HSC70, USP14, USP7, USP25, USP10, TRIM26 and GAPDH proteins.

### Tumor xenograft study

All animal manipulations were conducted in accordance with the guidelines and under the review and approval of the Animal Care Welfare Committee of Lanzhou University Second Hospital (Approval No. D2024-011). To establish the xenograft model, male nude mice (6–8 weeks old) were obtained from GemPharmatech and injected subcutaneously with DU145 cells (5 × 10^6^) into the flank. Once the tumors reached an approximate volume of 100 mm^3^, these mice were randomly assigned into three groups (n = 6 per group) and were intraperitoneally injected with PBS, evodiamine (10 mg/kg), or evodiamine (20 mg/kg) every two days for two weeks. After 7 cycles of drug administration, the mice were sacrificed, and the tumors were collected. Tumor size was measured by using digital calipers, and the tumor volumes were calculated using the formula: width × width × length/2. The excised tumor specimens were subsequently subjected to comprehensive pathological and molecular analyses, encompassing H&E staining, immunohistochemical (IHC) staining, and Western blot.

### Histopathologic analyses

The excised xenograft tumors were fixed in paraformaldehyde overnight. Tumors were washed with PBS and dehydrated with ethanol followed by embedding, sectioning. H&E staining of tumor sections was performed. Immunohistochemical staining was also used to determine the level of Ki67. Images were obtained on an Olympus microscope.

### Statistical analysis

All statistical analyses were conducted using GraphPad Prism version 8.0 (GraphPad Software, Inc., SanDiego, CA, USA). Data were presented as the mean ± SD. Student’s t-test or one-way ANOVA was applied to analyze the differences between groups. P < 0.05 was considered statistically significant.

## Results

### Evodiamine suppresses cells growth, invasion and colony formation in prostate cancer cell

**Fig. 1:**
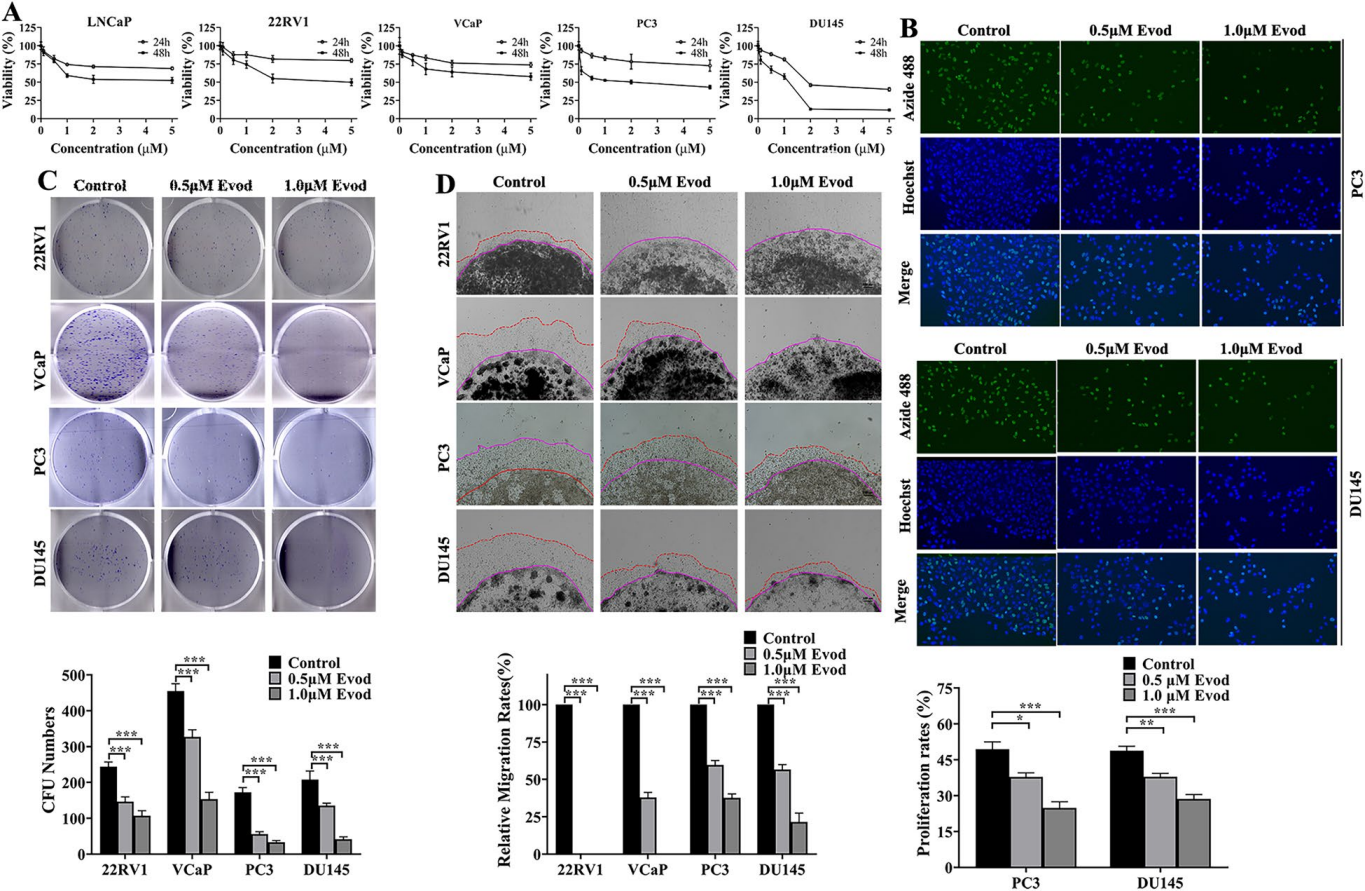
Evodiamine represses cell viability, invasion and colony formation in prostate cancer cells. **A** LNCaP, 22RV1, VCaP, PC3, and DU145 cells were treated with indicated concentration of evod (0, 0.5, 1.0, 2.0, 5.0 μM) for 24 h and 48 h, and cell viability was measured by CCK8 assay. **B** An EdU proliferation assay was performed on PC3 and DU145 cells treated with evod (0, 0.5, and 1.0 μM) at 24 h. **C** Representative images of cell colonies after 10 days treatment with evod (0, 0.5, 1.0 μM). **D** 3D matrigel drop invasion assay was conducted to evaluate the invasion ability in prostate cancer cells receiving evod treatment for 3 days. *P < 0.05; **P < 0.01; ***P < 0.001

Evodiamine is a natural product extractable from Evodia rutaecarpa, demonstrating the ability to inhibit tumor cell activity in various cancers, including breast cancer, colorectal cancer, and ovarian cancer. To examine the effect of evodiamine on the growth of prostate cancer cells, multiple prostate cancer cell lines, including castration-sensitive (LNCaP and 22RV1) and castration-resistant (VCaP, PC3 and DU145) prostate cancer cells, were treated with different doses of evodiamine for 24 and 48 h. A CCK8 assay was used to evaluate cell viability. Consistent with previous studies, evodiamine inhibited the activity of prostate cancer cells in a dose-dependent manner (Fig. 1A). Consistent with the cell viability assay, evodiamine inhibited the proliferation of PC3 and DU145 cells, as determined by the EdU thymidine analog incorporation assay (Fig. 1B). A CFU assay revealed that evodiamine treatment suppressed colony formation in 22RV1, VCaP, PC3, and DU145 prostate cancer cells (Fig. 1C). Additionally, a 3D matrigel drop invasion assay was employed to assess cell invasion capability. Our analysis showed that evodiamine treatment reduced the invasion ability of 22RV1, VCaP, PC3, and DU145 cells (Fig. 1D). In summary, evodiamine demonstrates the ability to inhibit proliferation and invasion in various prostate cancer cells. Therefore, evodiamine may serve as a promising compound of traditional Chinese medicine for anti-prostate cancer therapy.

### Evodiamine induces cell death in an apoptotic independent manner

**Fig. 2:**
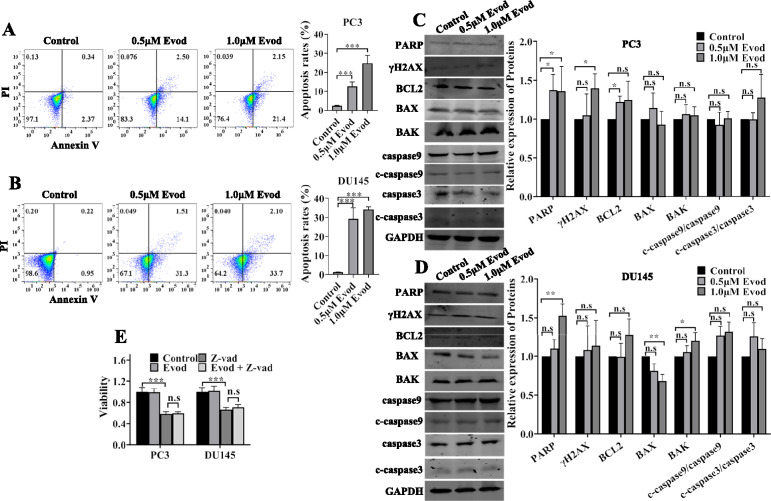
Evodiamine induces prostate cancer cell death in an apoptotic independent manner. **A** PC3 cells and **B** DU145 cells were treated with indicated concentration (0, 0.5, and 1.0 μM) of evod for 48 h, and the apoptosis rates were detected by flow cytometry. Apoptosis related proteins in PC3 (**C**) and DU145 (**D**) cells were analyzed by western blot with GAPDH as the loading control. **E** PC3 and DU145 cells were treated with 1.0 μM evod with or without Z-VAD-FMK (20 μM) for 48 h, after which cell viability was assessed using the CCK8 assay. *P < 0.05; **P < 0.01; ***P < 0.001

Multiple studies have demonstrated that evodiamine possesses the ability to trigger both apoptotic cell death and ferroptosis in tumor cells [22–25]. To further elucidate the mechanisms underlying the impact of evodiamine on prostate cancer cells, we initially conducted a PI/annexin V staining analysis. Our findings revealed that evodiamine treatment effectively induced cell death in PC3 and DU145 cells (Fig. 2A, B). Subsequently, Hoechst staining was performed to investigate the influence of evodiamine on the apoptotic bodies formation. Intriguingly, the presence of apoptotic bodies in prostate cancer cells was not significantly elevated following evodiamine treatment (Fig. S1). This observation aligns with the following findings that the ratio of cleaved caspase3 protein/ total caspase3 remained unaltered under the treatment of evodiamine (Fig. 2C, D). Z-VAD-FMK is a pan-caspase inhibitor capable of reversing cell death triggered by apoptosis. Our research further revealed that Z-VAD-FMK treatment failed to rescue prostate cancer cells from evodiamine-induced cell death (Fig. 2E). Collectively, these findings imply that evodiamine induces prostate cancer cell death through a mechanism independent of apoptosis.

### Ferroptosis contributes Evodiamine induced-cell death in prostate cancer cell

**Fig. 3:**
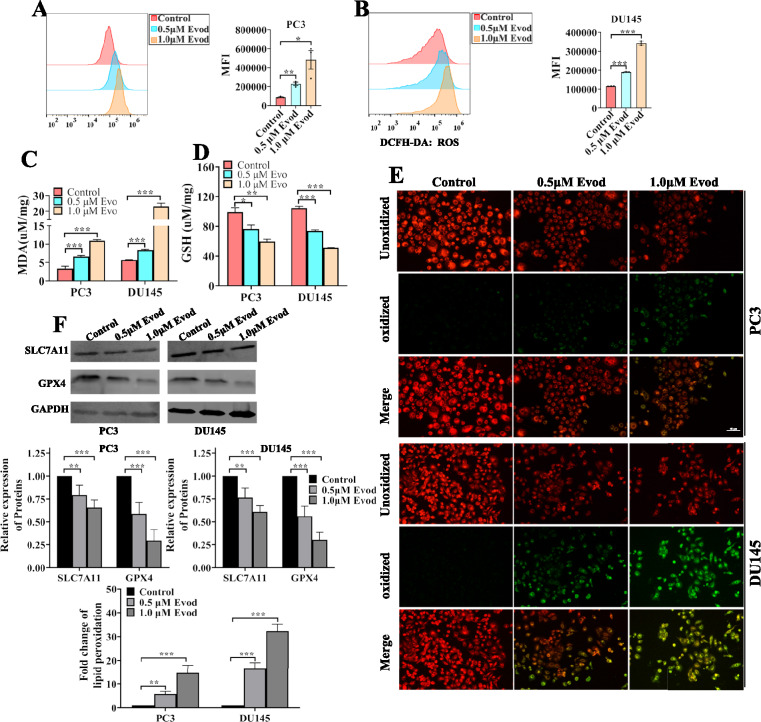
Ferroptosis contributes to Evodiamine induced-cell death in prostate cancer cells. PC3 and DU145 cells were treated with evod at concentrations of 0, 0.5, and 1.0 μM for 48 h. Following treatment, intracellular ROS (**A**, **B**), MDA (**C**), GSH (**D**) level were detected, the protein level of SLC7A11 and GPX4 were assessed using western blot with GAPDH as the loading control (**E**), and lipid peroxidation was probed by C11-BODIPY.^581/591^ (**F**). *P < 0.05; **P < 0.01; ***P < 0.001

Ferroptosis, a newly discovered form of cell death, induces cellular demise by regulating lipid peroxidation of cell membranes [8]. Recent studies have demonstrated that various alkaloids can induce cell death by activating the ferroptosis pathway [11]. Several research groups have reported that evodiamine induces apoptosis in tumor cells [21, 23]. Recent research has further shown that ferroptosis contributes to evodiamine-induced cell death [12, 24]. However, the specific role of ferroptosis in evodiamine-induced prostate cancer cell death remains unclear. In this study, we found that blocking ferroptosis with fer-1 or DFO in evodiamine-treated cells resulted in increased cell survival rates, indicating the involvement of ferroptosis in evodiamine-induced prostate cancer cell death (Fig. S2). Our findings also reveal that evodiamine treatment up-regulates ROS levels in prostate cancer cells (Fig. 3A, B). Moreover, compared to the control group, evodiamine-treated cells exhibited elevated levels of MDA and reduced levels of GSH (Fig. 3C, D). Additionally, staining with C11 BODIPY, a lipid ROS indicator, confirmed that evodiamine treatment increases lipid ROS levels (Fig. 3E). The xCt/GPX4 signaling pathway inhibits cell ferroptosis by enhancing GSH-mediated reduction of lipid peroxides. Herein, we observed that evodiamine inhibits GPX4 protein expression and slightly down-regulates SLC7A11 expression (Fig. 3F). Therefore, we conclude that evodiamine induces ferroptosis in prostate cancer cells.

### ROS mediates evodiamine-induced ferroptosis

**Fig. 4:**
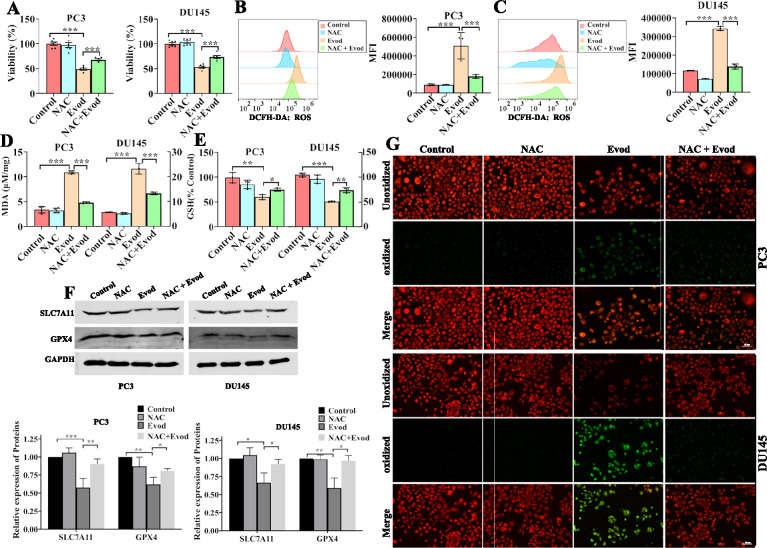
ROS scavenger inhibits the evodiamine-induced ferroptosis in prostate cancer cells. Cells were pretreated with 5.0 mM NAC for 1 h prior to treatment with 1.0 μM evod for 48 h, Following treatment, cell viability were assessed using the CCK8 assay (**A**), ROS levels were probed with DCFH-DA (**B**, **C**), MDA and GSH levels were quantitatively analyzed using commercial kits (**D**, **E**), The protein levels of SLC7A11 and GPX4 were evaluated by Western blot analysis with GAPDH as the loading control (**F**), and lipid peroxidation was probed by C11-BODIPY.^581/591^ (**G**). *P < 0.05; **P < 0.01; ***P < 0.001

The upregulation of ROS production serves as a pivotal indicator of ferroptosis, and numerous studies have further established that elevated ROS levels is intricately linked to alkaloid-induced ferroptosis [11]. Our study concurs with these findings, demonstrating that evodiamine induces an upregulation of ROS levels in prostate cancer cells (Fig. 3A, B). To gain insights into the role of ROS in evodiamine-induced ferroptosis, we employed NAC, a widely used ROS scavenger, to treat prostate cancer cells. Cell viability assays revealed that NAC treatment partially reverses the evodiamine-induced cell death (Fig. 4A). Furthermore, results also demonstrated that NAC treatment partially inhibited the evodiamine-induced upregulation the levels of cellular ROS, lipid ROS, and MDA (Fig. 4B–D, G). Notably, scavenging ROS also led to increased levels of reduced GSH and GPX4 in prostate cancer cells treated with evodiamine (Fig. 4E, F). Taken together, these results clearly indicate that ROS mediates evodiamine-induced ferroptosis in prostate cancer cells.

### Evodiamine triggers ferroptosis via targeting GPX4

**Fig. 5:**
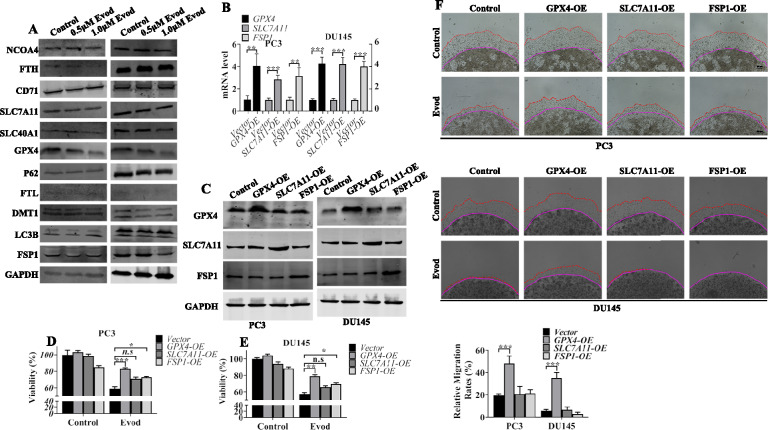
Evodiamine triggers ferroptosis via targeting GPX4. **A** The protein levels of NCOA4, FTH, CD71, SLC7A11, SLC40A1, GPX4, P62, FTL, DMT1, LC3B, and FSP1 were examined by Western blot analysis with GAPDH as the loading control in PC3 cells and DU145 cells treated with evod at concentrations of 0, 0.5, and 1.0 μM for 48 h. **B**, **C** mRNA and protein levels of *GPX4, SLC7A11* and *FSP1* measured by qRT-PCR and western blot in indicated cells. **D**, **E** Following a 48 h treatment with 1.0 μM evod, indicated cells’ viability was assessed using the CCK8 assay. **F** 3D matrigel drop invasion assay was used to assess the invasion capability of cells receiving 1.0 μM evod treatment or not. *P < 0.05; **P < 0.01; ***P < 0.001

**Fig. 6:**
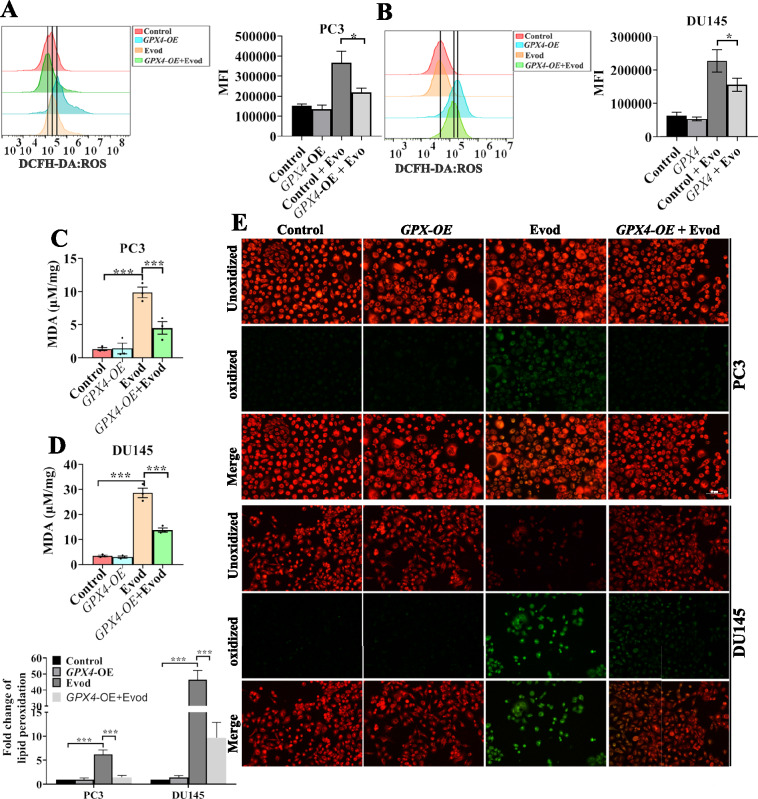
GPX4 overexpression reverses the inhibition of evodiamine in prostate cancer cells. **A**, **B** PC3 and DU145 cells were treated with 1.0 μM evod for 48 h. Subsequently, intracellular ROS levels were evaluated using the DCFH-DA probe. MDA levels was quantitatively analyzed using commercial kits (**C**, **D**), and lipid peroxidation was probed by C11-BODIPY.^581/591^ (**E**). *P < 0.05; **P < 0.01; ***P < 0.001

In addition to the xCt/GPX4 signaling pathway, FSP1 can inhibit ferroptosis by reducing COQ10 and vitamin K, thereby mitigating lipid peroxidation [26, 27]. Conversely, NCOA4 has the potential to induce ferroptosis in tumor cells by facilitating the degradation of FTH and promoting ferritinophagy within autophagosomes [28]. To understand whether these signaling pathways play a role in evodiamine-induced ferroptosis in prostate cancer cells, we analyzed these proteins levels in prostate cancer cells stimulated with evodiamine (Fig. 5A). Our findings revealed that the expression levels of ferritinophagy-related proteins and iron ion metabolism-related proteins did not exhibit uniform changes upon evodiamine treatment. In addition to the downregulation of GPX4 and SLC7A11, the protein level of FSP1 was also reduced in evodiamine-treated prostate cancer cells. To gain further insights into the roles of FSP1, GPX4, and SLC7A11 in evodiamine-induced ferroptosis, these proteins were over-expressed in PC3 and DU145 cells (Fig. 5B, C). The results demonstrated that ectopic expression of GPX4 rescued cells from evodiamine-induced cell death (Fig. 5D, E). Additionally, a 3D matrigel drop invasion assay revealed that overexpression of GPX4 significantly reversed the inhibitory effect of evodiamine on the invasion ability of prostate cancer cells (Fig. 5F). Collectively, these results suggest that evodiamine-induced ferroptosis in prostate cancer cells may not depend on regulating cellular iron metabolism, but rather on inhibiting the expression of the GPX4 protein. To further validate the pivotal role of GPX4 in evodiamine-triggered ferroptosis, we examined whether GPX4 overexpression could rescue the ferroptotic phenotypes. Results showed that overexpression of GPX4 partially suppressed evodiamine-induced ROS level (Fig. 6A, B), MDA production (Fig. 6C, D), and lipid ROS generation (Fig. 6E). These findings indicate that the downregulation of GPX4 expression indeed mediates evodiamine-induced ferroptosis in prostate cancer cells.

### Evodiamine treatments caused GPX4 protein instability by reducing TRIM26 expression

**Fig. 7:**
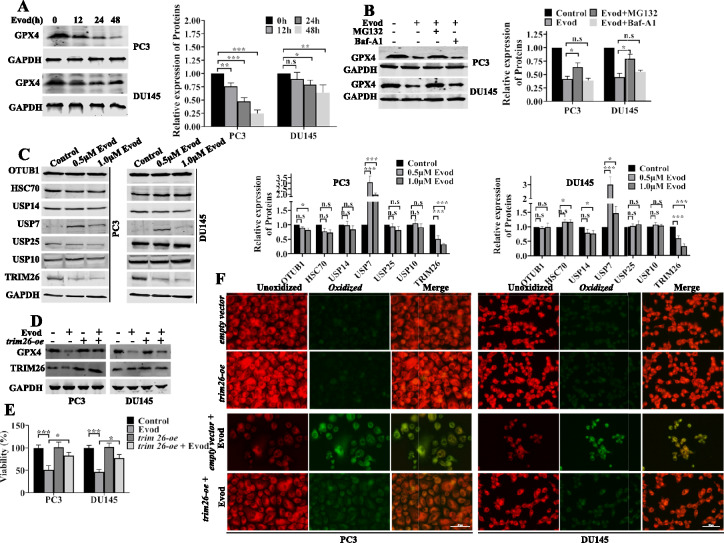
Evodiamine treatments caused GPX4 protein instability by reducing TRIM26 expression. **A** GPX4 protein levels were assessed by Western blot in PC3 and DU145 cells treated with 1.0 μM evod for 0, 12, 24 and 48 h. **B** PC3 and DU145 cells were treated with 1.0 μM evod, 10 μM MG132 or 200 nM Baf-A1 for 12 h, and GPX4 expression was measured by Western blot with GAPDH as the loading control. **C** The protein level of OTUB1, HSC70, USP14, USP7, USP25, USP10, and TRIM26 in PC3 and DU145 cells were evaluated following treatment with evod at concentrations of 0, 0.5, and 1.0 μM for 48 h. **D** The protein levels of GPX4 and TRIM26 were assessed in both control and *trim26*-overexpressing PC3 and DU145 cells, with or without a 48 h treatment with 1.0 μM evod. Following 48 h treatment with evod, cell viability was measured using the CCK8 assay (**E**) and the lipid peroxidation levels were investigated using C11-BODIPY.^581/591^ staining (**F**). *P < 0.05; **P < 0.01; ***P < 0.001

To unravel the mechanism underlying evodiamine-decreased GPX4 protein expression, we examined the mRNA expression levels of *GPX4* in PC3 and DU145 cells at various time points following evodiamine stimulation. Our results indicated that evodiamine treatment did not suppress *GPX4* mRNA expression (Fig. S3A). Consequently, we conducted a more detailed analysis of GPX4 protein expression levels in prostate cancer cells at different time points after evodiamine stimulation. Consistent with previous findings, we discovered that evodiamine can inhibit GPX4 protein expression in prostate cancer cells (Fig. 7A). Therefore, we hypothesize that evodiamine's effect on GPX4 expression may depend on its regulation of GPX4 protein stability. Autophagy and proteasomal degradation are pivotal in regulating protein stability. To delve into the mechanisms governing evodiamine-induced GPX4 protein instability, we utilized the proteasome inhibitor MG-132 and the autophagy inhibitor Baf-A1. Our results suggest that proteasomal degradation pathway is essential for regulating GPX4 protein stability in prostate cancer cells (Fig. 7B). Notably, proteins such as OTUB1, HSC70, USP14, USP7, USP25, USP10, and TRIM26 play crucial roles in regulating GPX4 degradation [28–31]. We conducted a comprehensive analysis of the levels of these proteins and explore their contributions to GPX4 protein stability (Fig. 7C). The findings revealed that TRIM26 expression decreased upon evodiamine stimulation. TRIM26 is capable of enhancing GPX4 protein stability by interacting with GPX4 and catalyzing its ubiquitination at K107 and K117, thereby promoting K63 polyubiquitination of GPX4. To gain deeper insights into the role of TRIM26, we overexpressed it in prostate cancer cells (Fig. S3B, C) and subsequently evaluated its influence on evodiamine-induced inhibition of GPX4 protein expression. Our results showed that TRIM26 overexpression partially reverses the evodiamine-induced suppression of GPX4 protein expression (Fig. 7D) and preserves cell viability under evodiamine treatment (Fig. 7E). Moreover, TRIM26 overexpression partially reduces lipid ROS production in these cells (Fig. 7F). These results suggest that evodiamine promotes ferroptosis in prostate cancer cells by inhibiting TRIM26 protein expression, thereby destabilizing GPX4 protein and inducing cell death.

### Effects of evodiamine on the DU145 xenograft model

**Fig. 8:**
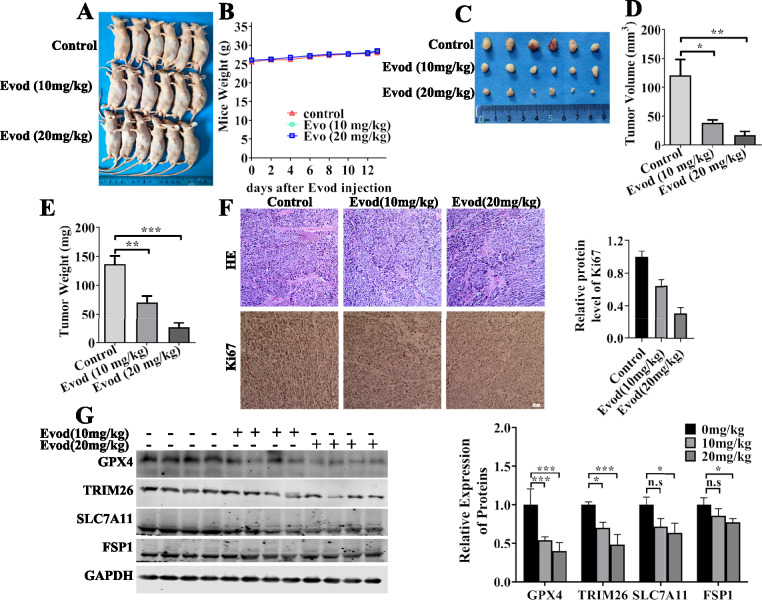
Evodiamine treatment suppresses cancer progression in DU145 xenograft model. Subcutaneous xenografts were established in nude mice by injecting DU145 cells into their flank. 10 days post-inoculation, the mice received intraperitoneal treatments with either evod at doses of 10 mg/kg and 20 mg/kg, or phosphate-buffered saline (PBS), administered every two days for a total of 12 days. **A** Photographic images of nude mice bearing xenograft tumors are presented. **B** The body weights of the mice were recorded throughout the experiment. **C** After euthanasia, tumor tissues were excised and photographed. **D** Tumor volumes and (**E**) tumor weights were subsequently calculated. **F** Representative images of H&E staining and IHC staining for Ki67 are shown. **G** Protein levels of GPX4, TRIM26, SLC7A11 and FSP1 were analyzed by western blot with GAPDH as the loading control. *P < 0.05; **P < 0.01; ***P < 0.001

Given that evodiamine can induce ferroptosis in prostate cancer cells in vitro through the TRIM26/GPX4 signaling axis, we evaluated its effects on prostate cancer tumor growth in vivo. Using a subcutaneous xenograft tumor model in nude mice, we found that, compared to the control group, the body weight of the mice in the evodiamine-treated group did not change significantly (Fig. 8B). Both tumor weight and tumor volume in the evodiamine-treated group decreased significantly compared to those in the PBS-treated group (Fig. 8C–E). IHC staining suggested that evodiamine treatment suppressed Ki67 expression in prostate tumors (Fig. 8F). Furthermore, analysis of protein expression in tumor tissues revealed that evodiamine consistently inhibited the expression of TRIM26 and GPX4 proteins, aligning with the in vitro findings (Fig. 8G). These in vivo findings suggest that evodiamine can inhibit prostate cancer tumor growth through the TRIM26/GPX4 signaling pathway.

## Discussion

Despite radical prostatectomy and radiotherapy, there are numerous candidate drugs available for the management of prostate cancer by targeting the androgen receptor, DNA damage repair, and other pathways [32–34]. Activating or inactivating these signaling pathways could trigger the apoptosis of tumor cells. For instance, the androgen receptor antagonist enzalutamide induces intrinsic apoptosis activation via increasing BAX expression, decreasing Bcl-2 expression, nuclear pyknosis, and genomic DNA fragmentation [35]. Blocking androgen receptor activation with androgen synthesis inhibitors could also promotes the apoptosis of prostate cancer cells in both androgen receptor positive and negative prostate cancer cells [36, 37]. Microtubule assembly damage by docetaxel also results activating the caspase pathway in prostate cancer cells [38, 39]. However, the escape from apoptosis is the most prevalent mechanism of drug resistance in prostate cancer cells. Studies have shown that the abnormal expression of several anti-apoptotic factors contributes to the prevention of drug-induced apoptosis in these cells [40–42]. Notably, recent studies have demonstrated that ferroptosis plays a crucial role in overcoming apoptosis resistance in cancer therapy. Ferroptosis, characterized by intracellular iron accumulation and lipid peroxidation, represents a promising therapeutic approach to overcome drug resistance in prostate cancer [43, 44]. Targeting ferroptosis could enhance the effectiveness of anti-androgen drugs and halt prostate tumor growth [10].

Recent studies have suggested that many natural compounds can inhibit tumor progression by inducing ferroptosis [11]. Evodiamine is a bioactive alkaloid derived from Evodia rutaecarpa. Previous study showed that it could inhibit prostate cancer cell proliferation and migration [45, 46]. Hu’s work demonstrated that evodiamine exhibits anti-cancer activity by suppression of GPX4 and induction of ferroptosis [24].

In this study, we aim to explore whether evodiamine also inhibits the growth of prostate cancer cells by inducing ferroptosis and to investigate how evodiamine triggers ferroptosis in prostate cancer cells. Consistent with previous studies, we found that evodiamine can inhibit the proliferation, colony formation, and invasion of prostate cancer cells. While some studies have shown that evodiamine induces apoptotic cell death in tumor cells [18, 21, 23], our research indicates that evodiamine does indeed induce prostate cancer cell death, but it does not occur through the apoptotic pathway. This is evidenced by the lack of increase in apoptotic bodies following evodiamine stimulation, as well as no changes in the levels of proteins associated with the apoptotic signaling pathway. Furthermore, the pan-caspases inhibitor z-VAD-fmk does not reverse the inhibitory effects of evodiamine on prostate cancer cell. To further investigate the molecular mechanisms by which evodiamine induces cell death in prostate cancer, we found that ferroptosis was involved in it. This is evidenced by the upregulation of lipid ROS and MDA levels induced by evodiamine, along with a decrease in intracellular GSH, SLC7A11, and GPX4 levels. The accumulation of ROS contributes to the process of ferroptosis [7, 8]. Our study also found that ROS are involved in mediating evodiamine-induced ferroptosis in prostate cancer cells, as treatment with NAC significantly inhibits evodiamine-induced ferroptosis. Consequently, we analyzed the molecular mechanisms underlying evodiamine-induced ferroptosis in prostate cancer cells. Our findings revealed that GPX4, but not SLC7A11 or FSP1, is involved in mediating evodiamine-induced ferroptosis. GPX4, a key regulator of ferroptosis, protects cells from lipid peroxidation by reducing lipid hydroperoxide [47]. Indeed, we found that the overexpression of GPX4 can reverse the ferroptosis induced by evodiamine. Our study also demonstrated that evodiamine treatment decreased GPX4 protein stability. We systematically analyzed the expression of proteins that may affect GPX4 protein stability, including OTUB1, HSC70, USP14, USP7, USP25, USP10, and TRIM26. Our findings indicate that TRIM26 plays crucial role in this process. Overexpression of TRIM26 can upregulate GPX4 protein level in evodiamine-treated cells, while can also inhibit lipid ROS production. Finally, xenografts derived from prostate cancer cell lines confirmed the effect of evodiamine in delaying tumor growth in vivo. IHC and Western blot analyses confirmed that evodiamine induces ferroptosis in prostate cancer cells by decreasing TRIM26 expression, which in turn inhibits the stability of GPX4.

In this study, we discovered that evodiamine can inhibit the activity of various prostate cancer cell types, including PCa and CRPC. This finding shows significant implications for overcoming clinical drug resistance in prostate cancer. Specifically, to elucidate the mechanism underlying evodiamine-induced ferroptosis, we are the first to demonstrate that evodiamine induces ferroptosis by inhibiting the expression of TRIM26. This inhibition decreases the stability of the GPX4 protein in cells, promoting its degradation and ultimately leading to ferroptosis. However, this study also has certain limitations, primarily regarding the lack of identification of the specific target of evodiamine and the relationship between the target and ferroptosis. In summary, these collective data suggest that targeting ferroptosis with evodiamine may represent a promising new strategy for the treatment of prostate cancer.

## Conclusion

In summary, our results illustrate that evodiamine exerts potent antitumor effects against prostate cancer through promoting TRIM26-mediated degradation of GPX4 protein and triggering ferroptosis. These findings suggest that evodiamine, a natural product derived from traditional Chinese medicine, could be a promising therapeutic agent for prostate cancer.

## Supplementary Information

Additional file 1. Fig. S1 Treatment with evodiamine has no effect on the content of apoptotic bodies in prostate cancer cells. The 22RV1, VCaP, PC3, and DU145 cells were treated with evod at concentrations of 0, 0.5, and 1.0 μM for a period of 48 h. Following this treatment, the cells were stained with Hoechst 33258. Representative images obtained from these assays are presented.

Additional file 2. Fig. S2 Evodiamine-induced cell death depends on ferroptosis. PC3 and DU145 cells were treated with evod (1.0 μM) with or without fer-1 (2.0 μM), and DFO (5.0 μM) for 48 h. Then, Cell viability was measured using CCK8 assay.

Additional file 3. Fig. S3 Evodiamine treatments caused GPX4 protein instability by reducing TRIM26 expression. (A) PC3 and DU145 cells were treated with 1.0 μM evod for durations of 0, 6, 12, and 24 h. Following this treatment, the levels of *GPX4* mRNA were analyzed using quantitative polymerase chain reaction (qPCR). (B-D) trim26 mRNA levels were analyzed by qPCR, and TRIM26 protein levels were examined by Western blot in control and trim26-overexpressing (*trim26*-oe) PC3 and DU145 cells.

## Data Availability

The data used to support the finding of this study are available on request.

## Abbreviations

Evod: Evodiamine
MDA: Malondialdehyde
GSH: Glutathione
H&E: Hematoxylin and eosin
IHC: Immunohistochemistry
ROS: Reactive oxygen species (ROS)
NAC: N-acetylcysteine
PCa: Prostate cancer
CRPC: Castration-resistant prostate cancer
ADT: Androgen deprivation therapy
CFU: Colony-forming-unit
PBS: Phosphate-buffered saline
qRT-PCR: Quantitative RT-PCR
BCA: Bicinchoninic acid
GPX4: Glutathione Peroxidase 4
FSP1: Ferroptosis Suppressor Protein 1
SLC7A11: Solute Carrier Family 7 Member 11
TRIM26: Tripartite Motif Containing 26
