# Supplementary figures and images for: Evodiamine induces ferroptosis in prostate cancer cells by inhibiting TRIM26-mediated stabilization of GPX4
Source: Chin Med. 2025 May 26;20:71. doi: 10.1186/s13020-025-01130-0 (PMC12105283; doi:10.1186/s13020-025-01130-0)

**22RV1****VCaP****PC3****DU145****Control****0.5 $\mu$ M Evod****1.0 $\mu$ M Evod**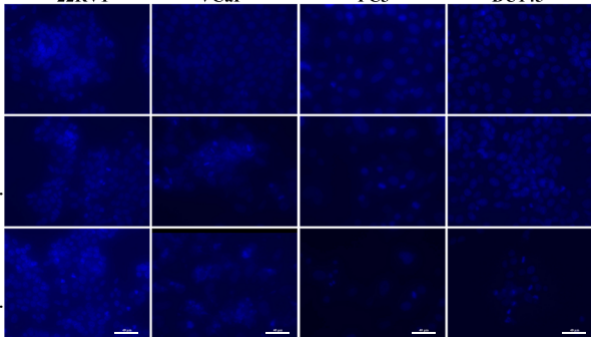

Supplement: Supplementary file 1 — Additional file 1. Fig. S1 Treatment with evodiamine has no effect on the content of apoptotic bodies in prostate cancer cells. The 22RV1, VCaP, PC3, and DU145 cells were treated with evod at concentrations of 0, 0.5, and 1.0 μM for a period of 48 h. Following this treatment, the cells were stained with Hoechst 33258. Representative images obtained from these assays are presented. [file 13020_2025_1130_MOESM1_ESM.pdf]

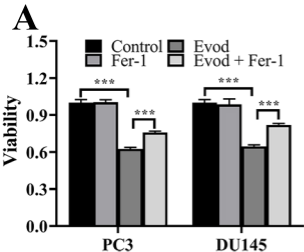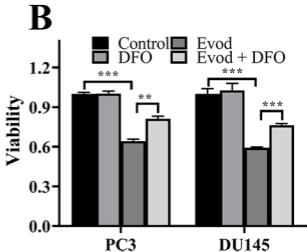

Supplement: Supplementary file 2 — Additional file 2. Fig. S2 Evodiamine-induced cell death depends on ferroptosis. PC3 and DU145 cells were treated with evod (1.0 μM) with or without fer-1 (2.0 μM), and DFO (5.0 μM) for 48 h. Then, Cell viability was measured using CCK8 assay. [file 13020_2025_1130_MOESM2_ESM.pdf]

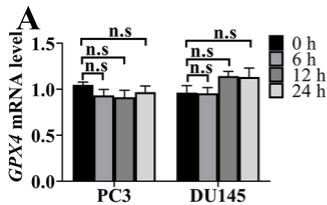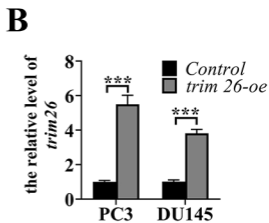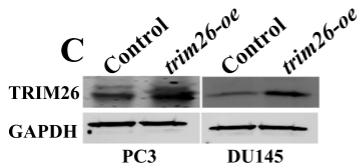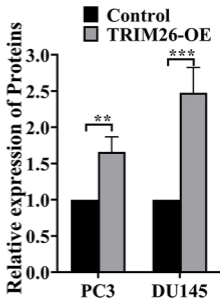

Supplement: Supplementary file 3 — Additional file 3. Fig. S3 Evodiamine treatments caused GPX4 protein instability by reducing TRIM26 expression. (A) PC3 and DU145 cells were treated with 1.0 μM evod for durations of 0, 6, 12, and 24 h. Following this treatment, the levels of GPX4 mRNA were analyzed using quantitative polymerase chain reaction (qPCR). (B-D) trim26 mRNA levels were analyzed by qPCR, and TRIM26 protein levels were examined by Western blot in control and trim26-overexpressing (trim26-oe) PC3 and DU145 cells. [file 13020_2025_1130_MOESM3_ESM.pdf]
